# Anomalous and heterogeneous characteristics of the BOLD hemodynamic response function in white matter

**DOI:** 10.1093/texcom/tgac035

**Published:** 2022-08-18

**Authors:** Kurt G Schilling, Muwei Li, Francois Rheault, Zhaohua Ding, Adam W Anderson, Hakmook Kang, Bennett A Landman, John C Gore

**Affiliations:** Vanderbilt University Institute of Imaging Science, Vanderbilt University Medical Center, Nashville, TN 37232, USA; Department of Radiology and Radiological Sciences, Vanderbilt University Medical Center, Nashville, TN 37232, USA; Vanderbilt University Institute of Imaging Science, Vanderbilt University Medical Center, Nashville, TN 37232, USA; Department of Radiology and Radiological Sciences, Vanderbilt University Medical Center, Nashville, TN 37232, USA; Department of Electrical Engineering and Computer Science, Vanderbilt University, Nashville, TN 37232, USA; Vanderbilt University Institute of Imaging Science, Vanderbilt University Medical Center, Nashville, TN 37232, USA; Department of Radiology and Radiological Sciences, Vanderbilt University Medical Center, Nashville, TN 37232, USA; Department of Electrical Engineering and Computer Science, Vanderbilt University, Nashville, TN 37232, USA; Vanderbilt University Institute of Imaging Science, Vanderbilt University Medical Center, Nashville, TN 37232, USA; Department of Radiology and Radiological Sciences, Vanderbilt University Medical Center, Nashville, TN 37232, USA; Department of Biomedical Engineering, Vanderbilt University, Nashville, TN 37232, USA; Department of Biostatistics, Vanderbilt University, Nashville, TN 37232, USA; Vanderbilt University Institute of Imaging Science, Vanderbilt University Medical Center, Nashville, TN 37232, USA; Department of Electrical Engineering and Computer Science, Vanderbilt University, Nashville, TN 37232, USA; Vanderbilt University Institute of Imaging Science, Vanderbilt University Medical Center, Nashville, TN 37232, USA; Department of Radiology and Radiological Sciences, Vanderbilt University Medical Center, Nashville, TN 37232, USA; Department of Biomedical Engineering, Vanderbilt University, Nashville, TN 37232, USA

**Keywords:** BOLD, fMRI, hemodynamic response function, white matter

## Abstract

Detailed knowledge of the BOLD hemodynamic response function (HRF) is crucial for accurate analyses and interpretation of functional MRI data. Considerable efforts have been made to characterize the HRF in gray matter (GM), but much less attention has been paid to BOLD effects in white matter (WM). However, several recent reports have demonstrated reliable detection and analyses of WM BOLD signals both after stimulation and in a resting state. WM and GM differ in composition, energy requirements, and blood flow, so their neurovascular couplings also may well be different. We aimed to derive a comprehensive characterization of the HRF in WM across a population, including accurate measurements of its shape and its variation along and between WM pathways, using resting-state fMRI acquisitions. Our results show that the HRF is significantly different between WM and GM. Features of the HRF, such as a prominent initial dip, show strong relationships with features of the tissue microstructure derived from diffusion imaging, and these relationships differ between WM and GM, consistent with BOLD signal fluctuations reflecting different energy demands and neurovascular couplings in tissues of different composition and function. We also show that the HRF varies in shape significantly along WM pathways and is different between different WM pathways, suggesting the temporal evolution of BOLD signals after an event vary in different parts of the WM. These features of the HRF in WM are especially relevant for interpretation of the biophysical basis of BOLD effects in WM.

## Introduction

Functional Magnetic Resonance Imaging (fMRI) based on blood oxygenation level-dependent (BOLD) contrast is well-established as a technique to map cortical activity in the brain. BOLD signals indirectly report neural activity and are characterized by a hemodynamic response function (HRF) which describes the effects of transient changes in blood flow, volume, and/or oxygenation (Boynton et al. 1996; Logothetis 2010). The HRF has been shown to vary in amplitude, timing, and shape across brain regions, cognitive states, with aging, and pathology (Handwerker et al. 2012; Badillo et al. 2013; West et al. 2019), yet accurate estimates of the HRF in any location are crucial for analyses and interpretation of fMRI data.

To date, most efforts to characterize the HRF have focused on measuring the transient, task-evoked BOLD responses to known events or stimuli (i.e. event-related fMRI), where timing information is accurately known. However, recent reports have shown how identification of the peaks of relatively large-amplitude BOLD signal fluctuations in resting-state data may also be used to reliably estimate HRFs without a stimulus (Tagliazucchi et al. 2012; Wu et al. 2013). This insight has led to the derivation and characterization of the HRF along the entire cortex in resting-state data (Wu et al. 2021), and the potential use of the HRF as a biomarker to study the effects of development, aging, or pathology (Wu and Marinazzo 2015; Rangaprakash et al. 2018; Yan et al. 2018).

Most measurements of the HRF have focused on gray matter (GM), as BOLD effects in white matter (WM) have been reported relatively rarely (Gawryluk et al. 2014; Gore et al. 2019) and often are regressed out as nuisance covariates (Grajauskas et al. 2019). Blood flow and volume in WM are only about 25% as large in GM (Rostrup et al. 2000; Helenius et al. 2003) and the energy requirements for WM functions are usually considered low compared with the cortex (Harris and Attwell 2012), so large BOLD effects are not expected. However, there have been several recent reports of successful detection and analyses of WM BOLD signals in both a resting state and after a task (Fraser et al. 2012; Li et al. 2019) (for a review see Gawryluk et al. 2014 and more recently Gore et al. 2019). These have led to increased awareness of the relationships between GM activity and WM BOLD signals and of the significant correlations between BOLD signals from different WM and GM regions in a resting state (Gao et al. 2021; Wang et al. 2021). Given the different composition, vasculature, and functions of GM and WM, their energy use and neurovascular coupling may be different. Characterizing the HRF in WM accurately is relevant for detection, quantification, and interpretation of BOLD effects.

The biophysical origins of BOLD signals in WM have not been identified, but 2 potential explanations include (i) they reflect venous drainage of deoxygenated blood from adjacent active GM or (ii) they correspond to increases in metabolic demand within WM that result from neural activity within GM and thus are similar to vascular effects in GM evoked by a stimulus. While neural signaling processes demand substantial energy consumption in GM (Rostrup et al. 2000; Helenius et al. 2003), the metabolic support of other processes is believed to dominate the energy budget in WM, in contrast to GM (Harris and Attwell 2012). These nonsignaling metabolic requirements include the maintenance of resting potentials on the membranes of cells such as oligodendrocytes, and the support of general housekeeping including the maintenance of myelin, and are a feature of energy-use in nonneuronal glial cells. Thus, BOLD signal fluctuations in WM may be driven by different energy demands and cell/tissue types than in GM, and the HRF may reflect the differences in neurovascular coupling. Recent reports have confirmed that WM HRFs in task-based fMRI are similar to but different from those in GM (Fraser et al. 2012; Li et al. 2019), with WM responses generally being lower in magnitude, delayed in time, and possibly featuring a more prominent initial dip. The initial dip could reflect the early arrival of venous drainage from GM or the initial decrease in tissue oxygenation that may occur prior to the increased inflow of arterial blood. We would predict that the former effect should be greatest in the WM immediately adjacent to GM, whereas the latter might be larger in deeper WM that is further from major arteries. Differences between GM and WM may reflect the different energy demands of the different cell types within GM and WM, their different vascular characteristics, and other variations in tissue microstructure such as cell densities within and between WM or GM regions.

We aimed to provide a comprehensive characterization of the HRF in WM across a population, including accurate measurements of its shape and its variation along and between WM pathways, using resting-state fMRI acquisitions. We also for the first time relate features of the HRF to microstructural and physiological properties of tissue derived from diffusion MRI. The relationships of HRFs between tissue types, and the measured differences in the HRF along and between WM pathways, suggest that there are different energy requirements and hemodynamic responses along different WM pathways which are consistent with the hypothesis that BOLD signals in WM reflect the metabolic needs of different tissue components than those seen in GM.

## Materials and methods

### Data

The data and HRF estimates closely followed the approach of Li et al. (2021). One hundred and ninety nine subjects were randomly selected from the HCP S1200 release (87 M/112 F; age 22–35). The images included resting-state fMRI, *T*_1_-weighted MRI, and diffusion MRI. The imaging protocols have been described in detail in previous reports (Van Essen et al. 2012). Briefly, data were acquired using a 3 T Siemens Skyra scanner (Siemens AG, Erlanger, Germany). The resting-state data were acquired using multiband gradient-echo echo-planar imaging (EPI). Each session consisted of 2 runs (with left-to-right and right-to-left phase encoding) of 14 min and 33 s each (TR = 720 ms, TE = 33.1 ms, voxel size = 2 mm isotropic, number of volumes = 1,200). Physiological data, including cardiac and respiratory signals, were recorded during fMRI acquisitions. The diffusion MRI were acquired using a multiband spin-echo EPI sequence, again with right-to-left and left-to-right phase encoding polarities (TR = 5520 ms, TE = 89.5 s, voxel size = 1.25-mm isotropic, 3 shells of *b* = 1,000, 2,000, and 3,000 s/mm^2^). *T*_1_-weighted images were acquired using a 3D magnetization-prepared rapid acquisition with gradient echo (MPRAGE) sequence (TR = 2400 ms, TE = 2.14 s, voxels size = 0.7-mm isotropic).

### Preprocessing

Images were preprocessed through the minimal preprocessing (MPP) pipelines (Glasser et al. 2013) of the HCP. *T*_1_-weighted images were nonlinearly registered to MNI space using FNIRT (Jenkinson et al. 2012) and subsequently Freesurfer produced surface and volume parcellations as well as morphometric measurements (Dale et al. 1999). For fMRI, the analysis pipeline included motion correction, distortion correction using reversed-phase encoding directions, and nonlinear registration to MNI space. We performed additional processing including regression of nuisance variables, including head movement parameters (using one of the outputs of motion correction in the MPP pipeline), and cardiac and respiratory noise modeled by the RETROICOR approach (Glover et al. 2000), and followed by a correction for linear trends and temporal filtering with a band-pass filter (0.01–0.08 Hz). A group-wise WM mask was reconstructed by averaging the WM parcellations that were derived from Freesurfer across all subjects and thresholded at 0.9. A GM mask was reconstructed in a similar manner but using a lower threshold (0.6) due to higher individual variabilities in GM. For diffusion images, the MPP pipeline included a zero-gradient intensity normalization, EPI distortion correction using reversed-phase encoding directions, and, again, nonlinear registration to MNI space.

**Fig. 1:**
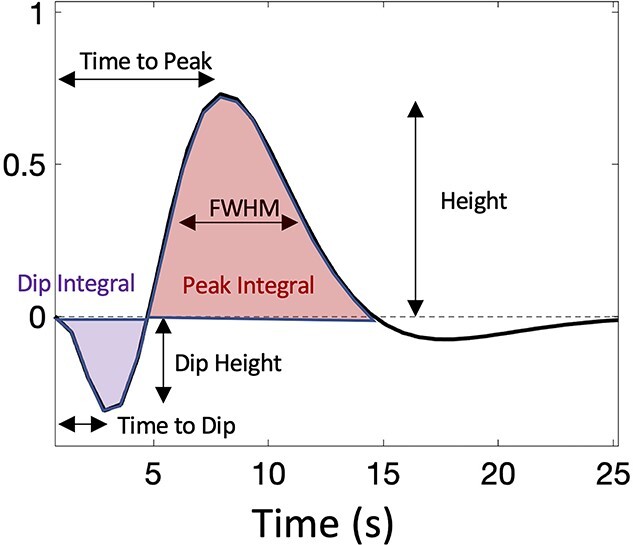
The HRF and its features: time to peak, height, FWHM, peak integral, time to dip, dip height, and dip integral.

**Fig. 2:**
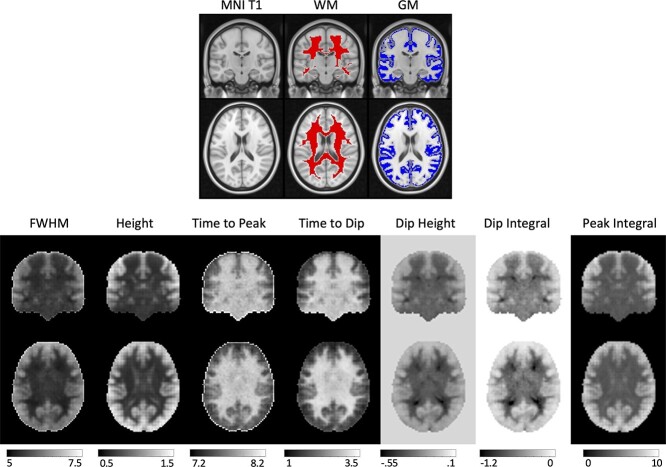
Population maps of HRF features show qualitative differences between GM and WM. (Top) shown are the MNI T1, WM, and GM masks for anatomical reference. (Bottom) population-averaged features of the HRF are shown for FWHM, height, PSC, time to peak, time to dip, and dip height.

### HRF estimation

HRFs were estimated from resting-state time courses in each subject using a blind deconvolution approach (Wu et al. 2013; Wu and Marinazzo 2015) implemented using the rsHRF toolbox (Wu et al. 2021). The method requires no prior hypothesis about the HRF and is based on the notion that relatively large amplitude BOLD signal peaks represent the occurrence of separable, major, spontaneous events. In our study, such events were detected as peaks beyond a specified threshold (here, greater than 1.5 standard deviations over the mean). For each event, a general linear model was fitted using a linear combination of 2 double gamma functions together with a temporal derivative to fit the derived waveforms. The double gamma functions together with temporal derivative are capable of modeling an initial dip, a time delay, and a later undershoot in the response (Friston et al. 1995, 1998).

### HRF features

After the HRF had been estimated for each voxel in MNI space, features of the HRF were extracted and visualized as separate parametric maps as shown in Fig. 1. These included (i) the full width at half maximum (FWHM), a measure of BOLD response duration in seconds; (ii) the peak height (Height), a measure of maximum normalized signal response; (iii) the Time to Peak, a measure of response latency in seconds; (iv) the Time to Dip (where evident), the time to reduce from baseline to the most negative early response; (v) the Dip Height (where evident), corresponding to the maximum normalized signal decrease from baseline (recorded as a negative value); (vi) the Peak Integral, the area under the curve of the positive BOLD response; and (vii) the Dip Integral, the area under the curve of the early negative BOLD response.

### Microstructure features

Diffusion MRI scans were used to derive approximate measures of average tissue microstructure, using Diffusion Tensor Imaging (DTI) and the Neurite Orientation Dispersion and Density Imaging (NODDI) technique (Zhang et al. 2012).

DTI characterizes the magnitude, degree of anisotropy, and orientation of directional diffusion. From this, measures of fractional anisotropy (FA), mean diffusivity (MD), axial diffusivity (AD), and radial diffusivity were derived. The measures are sensitive to a variety of microstructural features. For example, FA may reflect the coherence of membranes and restrictions, whereas RD is sensitive to myelination and axonal number and packing density (Song et al. 2005; Wheeler-Kingshott and Cercignani 2009). The NODDI model represents the signal in each voxel as the sum of 3 tissue compartments—intraneurite (sometimes called intracellular), extraneurite, and cerebral spinal fluid. The intraneurite compartment is composed of neurites (modeled as zero-radius sticks) with a distribution of directions that includes both an average direction and a spread of orientations around that direction. Thus, application of NODDI produced a set of parametric maps, averaged across the population, of (i) an isotropic volume fraction (ISOVF), (ii) the intraneurite volume fraction (or neurite density index; NDI), and (iii) an orientation dispersion index where a higher value represents a larger spread of axon orientations. NODDI fitting on each voxel and subject was performed using the accelerated microstructure imaging via complex optimization (AMICO) method (Daducci, Canales-Rodriguez, et al. 2015).

Of particular importance, the quantity (1-NDI) represents the relaxation weighted volume fraction of all nonneurite components, which includes anything that does not display stick-like diffusion signal properties such as glial cells and some extracellular spaces.

### Along-tract quantification

To investigate HRF features of specific WM pathways, we analyzed a set of expertly delineated bundles in MNI space (Yeh et al. 2018). For this work, we focused on 15 major association, projection, and commissural pathways of the brain: the arcuate fasciculus (AF, left and right), corticospinal tract (CST, left and right), inferior longitudinal fasciculus (ILF, left and right), superior longitudinal fasciculus (SLF, left and right), optic radiation (OR, left and right), frontal aslant tract (FAT, left and. right), uncinate fasciculus (UF, left and right), and the corpus callosum.

HRF features were quantified along each major WM pathway using the along-fiber quantification technique on the population averaged WM tracts defined by Yeatman et al. (2012). Each pathway was segmented into *n* = 20 points. Along-pathway mean and standard deviations of each HRF feature were derived for each position along the pathway.

### Statistical analysis

Linear mixed-effects modeling was used to ask whether HRF features change along pathways: the regression equation used for each feature, y, was

$$ \mathrm{y} \!= \!\beta 0+{\beta 1}{\ast}\mathrm{position}+{\beta 2}{\ast }{\mathrm{position}}^2+{\beta 3}{\ast }{\mathrm{position}}^3+\beta 4\left(1|\mathrm{subject}\right) \!, $$

where subject was considered as a random effect (i.e. allowing for a subject-specific intercept), and we tested the null hypothesis that all fixed-effect coefficients equal zero (β1 = β2 = β3 = 0) using an F-test. A rejection of the null hypothesis suggests that a feature, y, changes along a pathway (note this tests whether there is significant change along a pathway but does not query which individual locations are different). We additionally performed a one-way analysis of variance (ANOVA) to test whether there were significant differences between pathways. We also evaluated whether the variation of each feature is different along each pathway, where the variation is defined as the ratio of the minimum to the maximum value of the feature along the pathway. All statistical tests were corrected for multiple comparisons (15 pathways × 7 features).

Finally, to assess relationships between different HRF features or between HRF and microstructural metrics, we performed 3 analyses (i) for each pair of features, we plotted one feature against the other for all WM and all GM voxels in MNI space, (ii) calculated the slope of line of best fit using total least squares regression (which minimized fitting error in both dependent and independent variables), and (iii) calculated the correlation coefficient between each pair of variables, again using all voxels in MNI space. Our primary purpose was to establish whether these relationships differ between GM and WM.

## Results

The results provide information in response to several questions we sought to address.

### What is the average HRF across a population?

Figure 2 shows WM and GM masks, and parametric maps of each of the 7 features of the HRF averaged across the population. WM and GM HRFs are qualitatively different, and all features show significant contrast between the tissues.

**Fig. 3:**
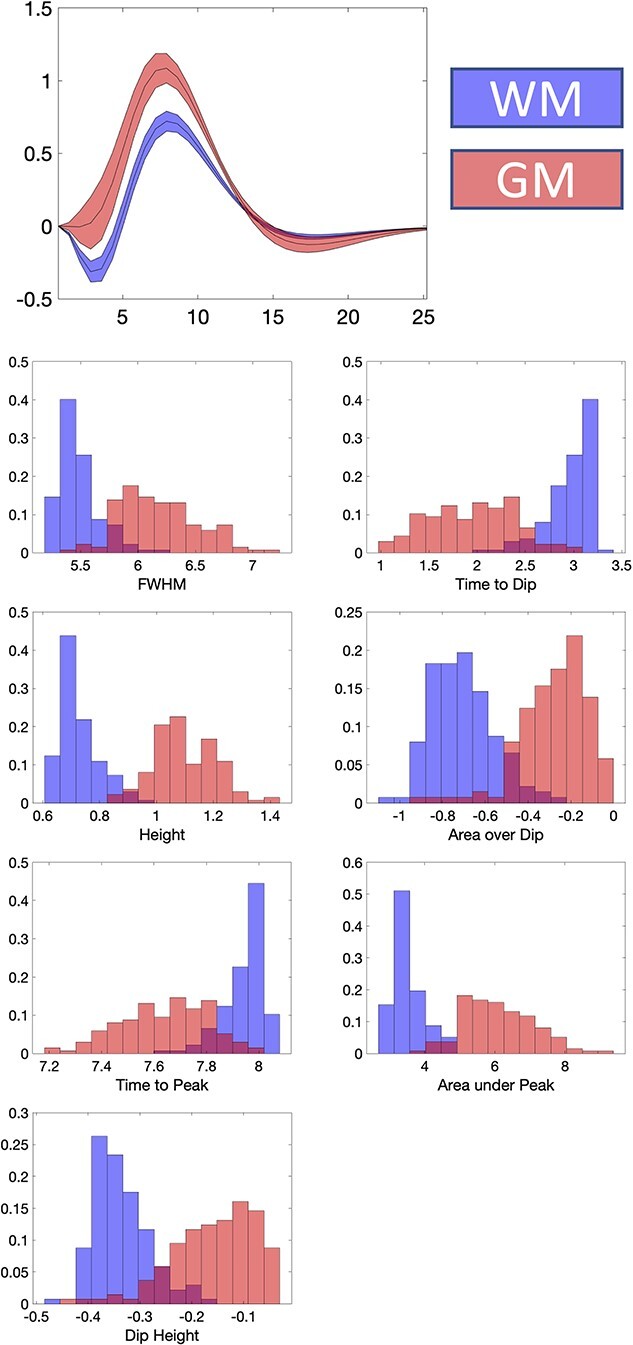
WM and GM have different HRFs and derived features. The population-averaged HRF is shown for WM and GM, displayed as mean and standard deviation across subjects. The distributions of HRF features in WM and GM are displayed as bar plots.

Figure 3 quantifies differences in each of the features of the HRFs between WM and GM, and the distributions of values within each tissue. The GM HRF shows a typical canonical response, with a barely evident initial dip, whereas the WM shows a consistent and larger initial Dip Height, a smaller Peak Height, and smaller negative undershoot. In general, WM HRFs have a shorter FWHM, lower Peak Height, longer Time to Peak, and larger negative Dip Area, in agreement with previous literature (Wu et al. 2021). In summary, there are significant differences between the HRF in WM and GM.

**Fig. 4:**
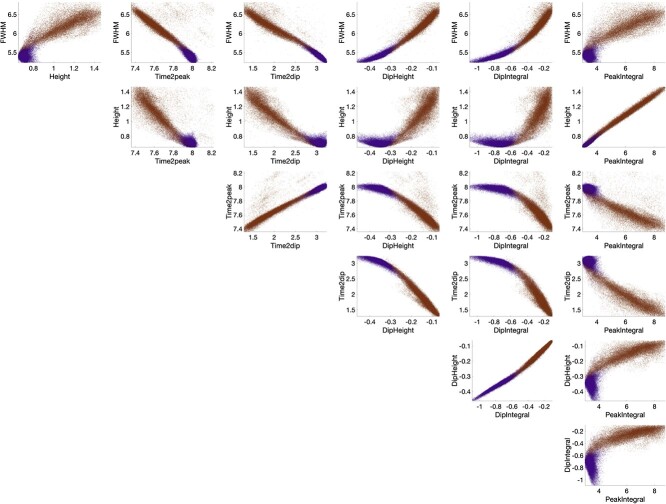
Features of the HRF show strong relationships with each other in both WM and GM. Here, each HRF features is plotted against all other HRF features for all voxels, with WM voxels shown as purple and GM as dark orange.

**Table 1:** Relationship between HRF features—Slope and correlation coefficient in both WM and GM. Slope in units of *y*-axis units/*x*-axis units.

		WM	GM	WM	GM	WM	GM	WM	GM	WM	GM	WM	GM	
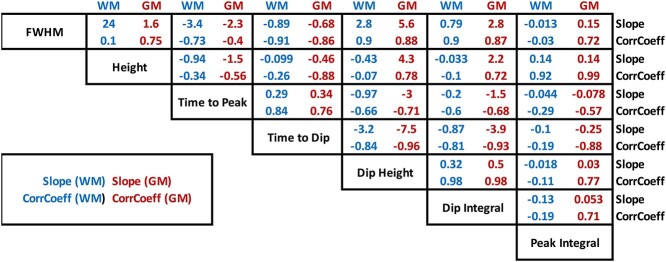														

### What are the relationships between HRF features?

Figure 4 plots the relationships between each pair of HRF features for all voxels in the population-averaged data, with WM voxels plotted as purple and GM as dark-orange. Calculated slopes and correlation coefficients of linear fits between each pair of HRF features are given in Table 1. Nearly, all features show strong correlations with others, and GM and WM exhibit similar trends but with some notable differences. Examples include strong positive relationships between time to peak and time to dip, dip height and dip integral, but with different slopes for GM and WM. In GM, a lower Dip Integral corresponds to a larger, faster positive BOLD increase (larger Height, FWHM, shorter Time to Peak) suggesting areas with a faster flow response to each event. Conversely, WM clearly exhibits larger Dip Integrals overall and the subsequent positive response shows little variation in Height or Time to Peak. In WM, there is little covariation of Peak Integral with FWHM, Peak Height, or Time to Peak, whereas in GM, the Peak Integral has strong positive correlation with the FWHM and Height and negative relationship with Time to Peak.

### What are the relationships between HRF and microstructure?

**Fig. 5:**
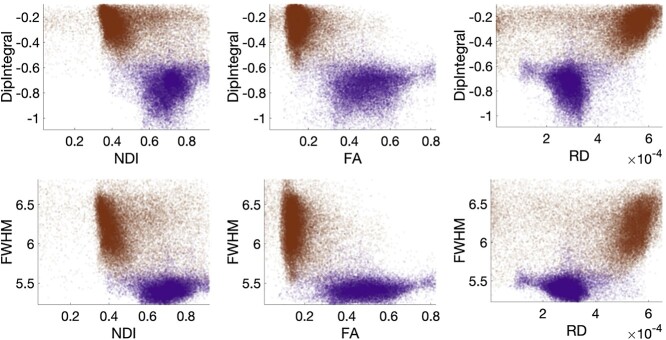
Features of the HRF show distinct relationships with tissue microstructure in WM and GM. Here, HRF features are plotted against microstructure features for all voxels, with WM voxels shown as purple and GM as dark orange.

Figure 5 plots the relationships between HRF features and diffusion-derived microstructural features for all voxels in the population-averaged maps. Again, WM and GM are shown as purple and dark orange, respectively. As expected, parameters describing tissue microstructure are different in WM and GM, and WM shows a higher FA, higher NDI, and lower RD. More interesting, WM and GM show very different relationships between the HRF and microstructure. For example, in WM, the Dip Integral (area under negative dip) has a smaller negative value with increasing NDI (*r* = +0.13) and with increasing FA (*r* = +0.11). However, this dip integral shows the opposite trend in GM, becoming more pronounced (larger negative values) with increasing NDI (*r* = −0.19) and increasing FA (*r* = −0.17). Likewise, the FWHM covaries with NDI and RD but with opposite relationships between WM (*r* = +0.11 for NDI, *r* = −0.07 for RD) and GM (*r* = −0.15 for NDI, *r* = +0.07 for RD). The slopes and correlation coefficients for relationships between all HRF feature and all microstructure features are given as Supplementary Information.

### How does the HRF vary along WM pathways?

**Fig. 6:**
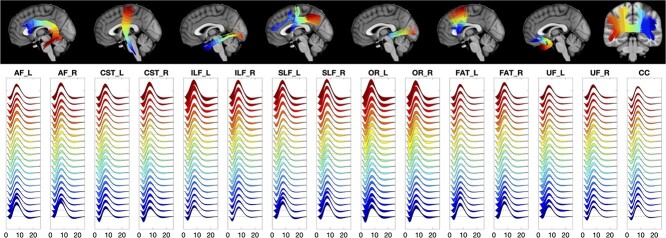
The HRFs are qualitatively different across and along pathways. Visualization of the HRFs along 15 WM pathways, color-coded from beginning (blue) to end (red) of pathways. Plots show the means and standard deviations across the population.

The HRFs for 15 WM pathways are shown in Fig. 6. Here, streamlines are color coded from blue to red, with corresponding color-matched HRFs shown averaged across the population. While the overall shape is similar within and across pathways, larger variations are observed near the WM/GM boundary at the ends of the pathway, with smooth trends along pathways. Visually, the peak heights decrease in the core of each WM pathway, whereas the dip height increases. Pathways such as the ILF, SLF, and OR show visually higher variation across the population.

**Fig. 7:**
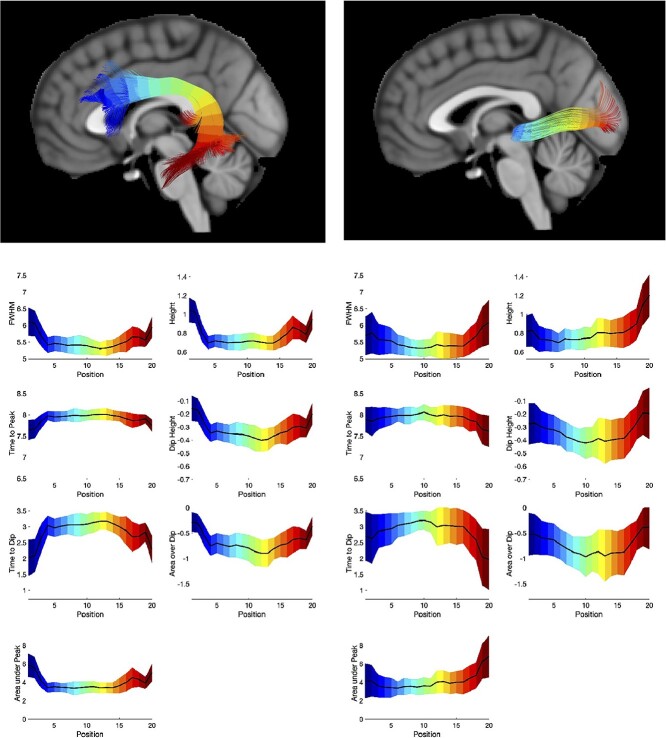
HRFs change along pathways. Exemplar pathways of the arcuate fasciculus and optic radiations are shown, with the 8 HRF features and their changes along the pathway. Color-coded to match the visualizations from beginning (blue) to end (red) of pathway. All features statistically significantly change along pathways.

Figure 7 shows two exemplar pathways (the arcuate fasciculus and optic radiations) and the corresponding changes in HRF features along each pathway. Trends are apparent along pathways, including a decreased height, decreased FWHM, increased time to peak and time to dip, and larger negative dip in the middle core of the pathway. Statistical analysis confirms that all features, of all pathways, significantly change along the pathway from start to end. All *P*-values for the *F*-tests are given as Supplementary Information.

### How does the HRF vary between WM pathways?

**Fig. 8:**
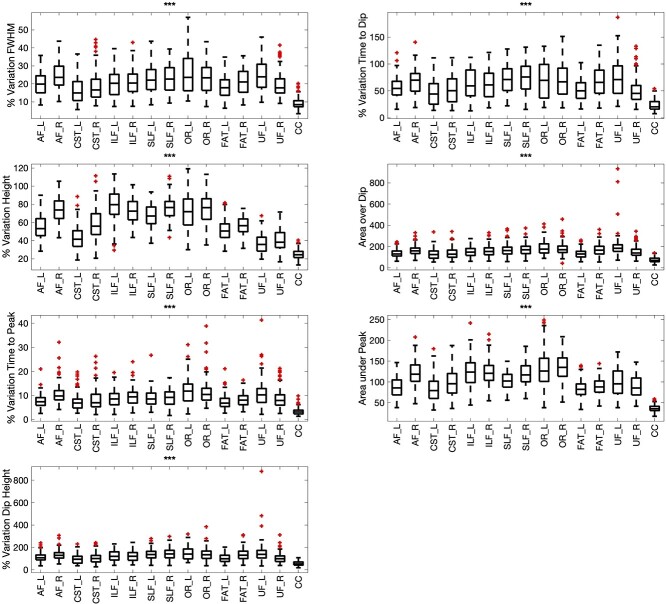
The variation along pathways (for each feature) is quantified by taking the range (max–Min) divided by the maximum value to indicate the “% variation” along pathways. This variation is shown for all pathways and is statistically significantly different between pathways.

We aimed to extract a measure of changes in each HRF feature along pathways by taking the ratio of the range of that feature over the maximum value, calling this “% Variation.” The % Variations of each feature, for all features along every pathway, are shown in Fig. 8. We find that several pathways show more variation in HRF features than others, including the optic radiations, and arcuate fasciculi, and that the observed variations are significantly different across all pathways.

## Discussion

We have quantified variations of the HRF of resting state fMRI within and across WM pathways and cortical GM by creating population-averaged parametric maps of HRFs and their derived features. We find that, following transient fluctuations in resting-state signal, WM BOLD responses have smaller peaks and are slower to change, than GM responses, and also feature a prominent negative initial dip. Second, we find strong links between different features of the HRF, with similar patterns observed in both WM and GM, suggesting similar factors affect the dynamics and evolution of the response function. However, the tissue microstructural environment differs significantly between these tissue types, and the dynamics of the HRF show different dependencies on the underlying structure. Next, we find that features of the HRF change significantly along WM pathways and are much different in the deep WM than at the superficial WM. The variation observed along pathways also differs between pathways. Altogether, this suggests that, much like in GM, changes in flow and/or oxygenation related to variations in baseline conditions are different for different parts of the WM. These differences in HRF features may be relevant for understanding the biophysical basis of BOLD effects in WM.

### Biophysical basis of BOLD signal in WM

The origins of BOLD effects in WM are not well understood though they share many features with BOLD effects in GM and thus a common explanation of their biophysical basis is plausible. The HRF features include an initial negative dip, indicating an increase in the concentration of deoxyhemoglobin within tissue, followed by a positive peak that extends for several seconds, indicating an increase in blood flow and volume, a net decrease or washout of deoxyhemoglobin, and then a return to equilibrium. The times for flow to increase and subsequently return to baseline are likely determined by the overall density and dimensions (diameters and spacings) of the vasculature involved (Bandettini et al. 1992; Buckner 1998; Logothetis et al. 2001). There is converging evidence that BOLD effects in WM are coupled with changes in neural activity within GM (Gore et al. 2019; Li et al. 2020; Guo et al. 2022), but it is not proven whether this reflects an intrinsic metabolic demand in WM or potentially flow effects from adjacent GM. The latter seems less likely given the nature of the vasculature within WM and the extent of the BOLD effects detected, though some features of the variations of HRF along tracts are consistent with a model in which blood draining from an activated region moves along a tract.

As pointed out by several previous reports (Harris and Attwell 2012; Yu et al. 2018), the metabolic demands of neural processes in WM and GM are different and only a relatively small fraction of the energy budget is WM that is believed to be required for signal transmission along axons. Thus, whereas in GM, the increase in oxygen consumption is directly related to intrinsic neural activity, in WM, the BOLD responses to activity within the cortex are likely triggered by a different requirement. Potential sources of increased metabolic demand include the glial and other non-neuronal cells that constitute a large fraction of WM (Stevens 2003). The 2 macroglia oligodendrocytes and astrocytes are the most abundant cell types. In addition to their roles in maintaining microstructure and production of essential lipids, glial cells are involved in a host of processes associated with brain function including the regulation of ionic balances, pH, neurotransmitter actions, and other requirements. If BOLD signals in WM are related to the metabolic requirements of the glial cells, we predict that there should be correlations between glial content and features of the HRF.

To investigate this potential connection, we produced atlases of microstructure features derived from diffusion MRI. From the diffusion models, it is possible to derive voxel-wise maps of parameters including FA and NDI. FA is highly sensitive to the fractional composition of tissue that is myelinated neurons. Complementing the neuronal fractional volume, the extracellular volume fraction (ECVF = 1 − NDI) is a measure of the volume fraction within a voxel that is not neuronal and thus is a surrogate metric of the glial cell volume fraction. Figure 5 shows the correlation between NDI and the negative dip of the HRF for WM and GM. If the area under the negative dip of the BOLD HRF indicates a larger metabolic demand within the tissue that causes tissue pO2 to decrease transiently, in WM, bigger negative dips (i.e. more negative dip integral) are related to lower NDI and hence greater glial cell composition. On the other hand, we find the inverse relationship between negative dip and ECVF in GM, consistent with the negative dip being smaller for cortex containing a smaller neuronal volume fraction.

### Variation along and between pathways

The use of diffusion tractography and along-fiber quantification (Yeatman et al. 2012) has proven useful in identifying location in the WM, and determining what changes occur, and where they occur, in developmental disorders, and disease. With the introduction of the rsHRF toolbox used here, Wu and colleagues noted differences in WM and GM HRF’s (Wu et al. 2021) and found areas in the WM that showed HRF alterations in schizophrenia and attention-deficit hyperactivity disorder. Here, we show that along-tract profiling is possible with nondiffusion-based measures and offers the ability to add specificity to identify which pathways may be affected and where along that pathway experiences alterations.

If the negative dip in WM reflects the passage of deoxygenated blood draining from activated cortex, and if the vasculature runs parallel to a tract, we would expect the times to dip and to peak to be progressively delayed along a tract, as found here. On the other hand, it is not clear on this model why the area under the dip should increase along a tract from both directions, and peak in the center.

In addition to showing feasibility of along-tract profiling of HRF features, our results also suggest that BOLD responses are different across different pathways in a resting state. This parallels the existence of resting-state networks in the cortex. Just as various networks may be characterized for aspects of attention, memory, motor, sensory systems, the WM fibers are structures often associated with unique functional roles, so it is unsurprising that they may require different energy processes on different time-scales. In fact, recent work (Wang et al. 2021) shows that by using appropriate analysis, the WM may be robustly parcellated into correlated networks. The use of a priori WM pathways, as opposed to these data-driven approaches, allows us to associate changes in blood flow and oxygenation to specific WM pathways with well-defined functional roles (Catani and Thiebaut de Schotten 2012).

There are several directions to pursue to extend this research. First, the use of the HRF as a potential biomarker would be strengthened if WM HRFs were shown to be changed in altered states/disorders as shown in GM (D'Esposito et al. 2003; Elbau et al. 2018; Yan et al. 2021), and if results are congruent with those suggesting deterioration of specific structural systems as studied with diffusion tractography (Yamada et al. 2009; Jbabdi and Johansen-Berg 2011). Studies like this would facilitate comprehensive evaluation and integration of structure and function. Second, HRFs can be quantified in task-based fMRI experiments. Experiments could be designed to elicit responses to study specific structure–function relationships, or for brain-wide activation (Taylor et al. 2018) that may facilitate normative pathway-specific responses during the signaling process. Similarly, relatively large amplitude BOLD signal peaks should be thoroughly characterized in WM, investigating frequency and clustering properties (Tagliazucchi et al. 2012; Petridou et al. 2013), relationship with low-frequency signal (Allan et al. 2015; Krishnan et al. 2018), and effects of fMRI preprocessing decisions (on both WM and GM) including smoothing, bandpass filtering, and motion correction. Finally, because pathways are known to spatially overlap in the brain, creating “crossing-fiber” (Jeurissen et al. 2013; Schilling et al. 2017) and “bottleneck” (Maier-Hein et al. 2017; Schilling et al. 2021) problems in the tractography process, it may be possible to disentangle multiple responses within the same voxel through a deconvolution process, or by using microstructure-informed or tractography-informed filtering (Daducci, Canales-Rodriguez, et al. 2015; Daducci, Dal Palu, et al. 2015; Girard et al. 2017; Barakovic et al. 2021), in order to extract multiple HRFs within the same WM voxel that could be analyzed on a fiber-element basis (Dhollander et al. 2021).

## Conclusion

We have characterized the HRF of resting state fMRI within and across WM pathways by creating population-averaged maps of the HRF and features of the HRF, and show that the HRF shows significant differences between WM and GM tissue types, features of the HRF show strong relationships with both other HRF features and with microstructural features, and that these relationships differ between tissue types. In particular, the dependences of some features of the HRF on neural content are reversed between GM and WM, suggesting that the BOLD response in WM may be related to different cellular types than in GM. We further find that the HRF varies significantly along pathways and is different between different WM pathways. Altogether, this suggests that, much like in GM, changes in flow and/or oxygenation related to variations in baseline conditions are different for different parts of the WM. These differences in HRF features may be relevant for understanding the biophysical basis of BOLD effects in WM.

## Supplementary Material

R1_Supplementary_Table_1_tgac035
